# Validation of the Spanish Center for Epidemiological Studies Depression and Zung Self-Rating Depression Scales: A Comparative Validation Study

**DOI:** 10.1371/journal.pone.0045413

**Published:** 2012-10-08

**Authors:** Paulo Ruiz-Grosso, Christian Loret de Mola, Johann M. Vega-Dienstmaier, Jorge M. Arevalo, Kristhy Chavez, Ana Vilela, Maria Lazo, Julio Huapaya

**Affiliations:** 1 Mental Health Working Group, Universidad Peruana Cayetano Heredia, Lima, Peru; 2 Hospital Nacional Cayetano Heredia, Lima, Peru; 3 Alberto Hurtado Medical School, Universidad Peruana Cayetano Heredia, Lima, Peru; Institute of Psychiatry at the Federal University of Rio de Janeiro, Brazil

## Abstract

**Background:**

Depressive disorders are leading contributors to burden of disease in developing countries. Research aiming to improve their diagnosis and treatment is fundamental in these settings, and psychometric tools are widely used instruments to support mental health research. Our aim is to validate and compare the psychometric properties of the Spanish versions of the Center for Epidemiological Studies Depression Scale (CES-D) and the Zung Self-Rating Depression Scale (ZSDS).

**Methodology/Principal Findings:**

A Spanish version of the CES-D was revised by 5 native Spanish speaking psychiatrists using as reference the English version. A locally standardized Spanish version of the ZSDS was used. These Spanish versions were administered to 70 patients with a clinical diagnosis of DSM-IV Major Depressive Episode (MDE), 63 without major depression but with clinical diagnosis of other psychiatric disorders (OPD), and 61 with no evidence of psychiatric disorders (NEP). For both scales, Cronbach's alpha (C-α) and Hierarchical McDonald Omega for polychoric variables (MD-Ω) were estimated; and receiver operating characteristics (ROC) analysis performed. For the CES-D and ZSDS scales, C-α was 0.93 and 0.89 respectively, while MD-Ω was 0.90 and 0.75 respectively. The area under the ROC curve in MDE+OPD was 0.83 for CES-D and 0.84 for ZSDS; and in MDE+NEP was 0.98 for CES-D and 0.96 for ZSDS. Cut-off scores (co) for the highest proportions of correctly classified (cc) individuals among MDE+OPD were ≥29 for CES-D (sensitivity (ss) = 77.1/specificity (sp) = 79.4%/(cc) = 78.2%) and ≥47 for ZSDS (ss = 85.7%/sp = 71.4%/cc = 78.9%). In the MDE+NEP, co were ≥24 for the CES-D (ss = 91.4%/sp = 96.7%/cc = 93.9%) and ≥45 for the ZSDS (ss = 91.4%/sp = 91.8%/cc = 91.6%).

**Conclusion:**

Spanish versions of the CES-D and ZSDS are valid instruments to detect depression in clinical settings and could be useful for both epidemiological research and primary clinical settings in settings similar as those of public hospitals in Lima, Peru.

## Introduction

Globally, unipolar depression is a major contributor of burden of disease, and its impact is growing [1], [2]. Also, research studies regarding risk factors, treatment, and associations of depressive disorders with other chronic and acute diseases show an increase on the importance of these mental disorders in global health and their harmful effect on people's health [3]–[5].

Depression scales such as the Beck Depression Inventory (BDI) [6], the Center for Epidemiologic Studies Depression Scale (CES-D) [7], the Zung Self-Rating Depression Scale (ZSDS) [8], and the Patient Health Questionnaire (PHQ) [9], [10] are widely used as depression screening tools for both diagnosis support and research. Recent meta-analytic studies report a similar performance in discriminative properties of the Hospital Anxiety and Depression Scale (HADS) (cut off score (co) ≥8; sensitivity (ss) = 0.82; specificity (sp) = 0.74) and of the Geriatric Depression Scale (GDS) (ss = 0.92; sp = 0.77) [11], [12]. A meta-analysis of Hamilton Rating Scale for Depression (HRSD), which is one of the most widely used, revealed that the intra class correlation coefficient was 0.94, the kappa coefficient 0.81, and the Pearson correlation coefficient 0.94 [13]. Current evidence suggests that there are no differences between them as screening tools for major depression [14] and their scores have showed good correlation [15].

The performance of various scales aiming to screen for depressive disorders has been reported with good results in Spanish language, including Latin America. For example, the Depressive Psychopathology Scale (DPS) reported good internal consistency (Cronbach's alpha (C-α) = 0.86) and discriminative power (ss = 77.7% and sp = 72.3) for the detection of major depressive disorder in a sample of Peruvian patients attending the National Institute of Mental Health [16]. Both 5 and 15 item versions of the GDS showed good psychometric properties in Spanish elderly (ss = 86.4/81.8%; 85.6/97.7%) [17], as well as an custom questionnaire for the elderly used in Mexico (C-α = 0.74; co≥5; ss = 80.7%, sp = 68.7%) [18] and the Edinburgh Postpartum Depression Scale (EPDS) in Puerperal Mexican Woman (C-α = 0.75; area under receiver operating characteristics curve (auROC) = 0.80, co≥7; ss = 75%; sp = 84%) [19].

In other Spanish speaking settings outside Latin America, the PHQ-15 showed a good internal consistency (C-α = 0.78), as did the GDS, when used in visually impaired individuals (C-α = 0.91) and the Postpartum Depression Screening Scale (C-α = 0.97) [20]–[22]. Adequate internal consistency and discriminative properties were reported for the World Health Organization Disability Assessment Schedule II (WHO-DAS II) (co≥50; ss = 71.4%; sp = 67.6%; proportion of correctly classified individuals (cc) = 70.4%) [22] and for the Euro-D in Spanish elderly (C-α = 0.75; area under the ROC curve auROC = 0.92, co≥3; ss = 91.8%; sp = 76.6%) [23].

The CES-D was found to be reliable in detecting depressive symptoms in Puerto Rican elderly with cognitive impairment (auROC = 0.90) [24], Colombian university students (C-α = 0.88) [25], adolescents from a sample of general population during a validation of short versions of 10 and 3 items (C-α = 0.86/0.74; auROC = 0.83/0.80; ss = 77.8/77.6% and sp = 74.1/70.7%) [26] and full scale (C-α = 0.85; auROC = 0.82; co≥23; ss = 73.3%; sp = 73.6%) [27]. Also, good internal consistency (C-α = 0.93) was found in Mexican adolescents of middle school [28]. In Spain, the CES-D was validated on a sample of patients with mood disorders (co≥16; ss = 95%; sp = 91%) [29] and non-psychiatric population (C-α = 0.89; co≥26; ss = 90.6%; sp = 91.8%) [30]. A 10 item version of the ZSDS was validated in Colombian general population (C-α = 0.80; ss = 95.5%; sp = 70.3%) [31] and in Spanish primary care patients (ss = 95%; sp = 74%) [32]. Finally in general population from Puerto Rico the ZSDS showed good internal consistency (C-α = 0.85) [33].

In 2009, *Reuland et al* reviewed the diagnostic accuracy of Spanish language depression screening instruments. Only, three studies from non-US Spanish were selected by the authors based on quality appraisal (EDPS, MHAS and PHQ-9). The overall conclusion was that, based on their review (including US-based validations), the CES-D and the PRIME-MD-9 might be useful for detecting depressed patients in primary care on the United States of America. Despite this, there was little evidence on primary care performance of depression scales in non-US Spanish speakers [34].

The availability of free to use, valid instruments might encourage independent research initiatives addressing the diagnosis, treatment and prevention of MDE in low and middle income countries, as well as contribute to alleviate the under diagnosis of depressive disorders in primary care [35]. Besides the DPS, which have showed to be appropriate for the discrimination of MDE on a population with a high prevalence of psychiatric disorders, such as the Peruvian National Institute of Mental Health population, few studies have studied the psychometric properties of depression screening scales in general hospital settings.

Both the CES-D and ZSDS scales have already been validated on university and middle school adolescents, elderly and general population in countries of Latin America; however, we could not find studies that specifically validated these scales on general hospital settings. We choose to validate the CES-D and ZSDS scales as both of them have already been validated in Latin America, their use poses no cost for researchers and there is evidence supporting their use albeit at different settings [28], [29], [33], [36]. This study aimed to evaluate the internal consistency and validity of an adapted Spanish version of the CES-D and ZSDS scales for the detection of Major Depressive Episode (MDE) using the diagnosis of a psychiatrist as defined by DSM-IV criteria as reference, and to compare the psychometric properties of the adapted scales.

## Methods

### Objectives

This study aimed to estimate the internal consistency and cutoff points for a maximum number of persons correctly classified as well as highest sensitivity and specificity of the CES-D and ZSDS on a sample of participants with clinical diagnosis of MDE, other mental disorders and persons without evidence of mental disorders on waiting room of a public hospital in a developing country. Discriminative properties of both scales are also compared.

### Participants

All of the patients who were waiting for services at the psychiatric and internal medicine outpatient consultations at the “Hospital Nacional Cayetano Heredia” (HNCH) from January to December 2006 were invited to participate in the study. The HNCH is a third level public hospital that serves three of the most populated districts in the northern part of Lima. Mental health care in Peru is usually provided in the psychiatry outpatient consultation areas in hospitals like HNCH. All patients who participated spoke Spanish as their native language. Patients attending HNCH are usually of low socio-economic status and participants were between 18 and 65 years of age. In terms of the groups of participants, those recruited from the psychiatry consultation included 70 people with Major Depressive Episode (MDE) and 63 people with other DSM-IV diagnostics (OPD), and those recruited from the internal medicine outpatient consultation included 61 people with no evidence of psychiatric disorders (NEP). Consecutive patients on the waiting room of the psychiatry and internal medicine consultation were invited to participate in the study until the calculated sample size for the study was achieved. Everyone gave informed consent prior to initiating participation. MDE diagnosis by a psychiatrist using DSM-IV criteria was an inclusion criterion for the MDE group, while the DSM-IV diagnosis of other psychiatric disorders but not MDE by the same psychiatrists was a criterion for the OPD group. We ruled out major depressive episode in the NEP group using the DSM-IV Structured Clinical Interview for Major Depression (SCID-I), and excluded those that resulted positive for MDE. We also excluded patients with physical or mental pathologies that could prevent them from completing the psychometric tests. Literacy was checked as part of the informed consent process by the investigators.

For the sample size calculation, a sensitivity and specificity of 80% was assumed, and the minimal acceptable value was 70% for both. To calculate the sensitivity and specificity with a confidence interval of +/−10 and a probability of Type I error of 5%, we needed to recruit at least 61 participants in each group (MDE, OPD and NEP).

### Study Design

A cross sectional design was used to establish the validity, internal consistency, and psychometric differences of the Spanish versions of CES-D and the ZSDS. Three groups of participants were established, one with no evidence of psychiatric disorders (“NEP” group), one with major depressive episode (“MDE” group), and the other without major depressive episode but with any other psychiatric disorder (“OPD” group). A sample of outpatients from the psychiatric and internal medicine consultations from the Hospital Nacional Cayetano Heredia (HNCH) in Lima, Peru was used to establish these groups.

### Data Collection

All of the groups of participants completed the pen and paper versions of the CES-D and ZSDS while on the waiting room of medicine or psychiatry outpatient consultation, and their socio-demographic data was also registered by the investigators. The psychiatric diagnosis, based on the DSM-IV classification, any other medical disease, and the Clinical Global Impression- Severity Scale (CGI-S) were made and registered by the medical assistant in charge of the consulting room, except in the NEP group where no CGI-S values were recorded.

### Instruments

The CES-D is a 20-item scale designed to be a case-detection instrument for depressive disorders in the general population. It has been validated in several languages, including Spanish [29], [37], [38], and in a wide range of populations, including adolescents [39]–[42] and elderly people [43]–[46]. Previous studies support the use of CES-D as a good psychometric test in cross-cultural contexts such as Latin America [47]. The CES-D is composed of 20 items; each one scored in a scale from “0” to “3” according to the amount of days on the previous days that the person felt according to the item's premise, thus the total score varies from 0 to 60.

The ZSDS is also a 20-item scale to help physicians in primary care settings to identify depressive symptoms [48], [49]. The ZSDS has been validated and its factor structure analyzed in different languages [50]–[52] and in diverse specific populations such as oncology patients [53], people with cognitive impairment [54] and Parkinson's disease [55], and college and university students [56], [57]. The ZSDS is also composed of 20 items, each one with punctuation from 1 to 4, making the range of the completed scale range from 20 to 80.

The CES-D scale used was the Spanish online version from the Center of Epidemiological Studies adapted by the Patient Education Center from the Medical School of Stanford University. It was revised by a group of 5 native Spanish speaker psychiatrists having as reference the English version provided by the same organization and a consensus version was reached. The psychiatrists suggested a few minor changes in the use of some words. The used ZSDS version corresponds to a standardized revised version used by the Peruvian National Mental Health Institute (PNMHI) in Lima.

### Statistical Methods

Statistically significant differences in age, educational level, gender and scores on the CGI, CES-D and ZSDS were analyzed across the MDE, OPD and NEP groups using a non-parametric test (Kruskall Wallis test and Spearman's rank correlation coefficient, “Rho”) since the data did not fulfill the normality assumption for the use of parametrical tests. Sensitivity and specificity were assessed for each scale independently using ROC curves, and the best cut-off points were determined using the score with the highest percentage of correctly classified individuals.

We also examined statistically significant differences in the area under the ROC curve between the CES-D and the ZSDS. ROC curve analyses were performed using the MDE versus the NEP (MDE+NEP), and then MDE versus the OPD (MDE+OPD). Differences between ROC curves were performed using a two sided hypothesis test of equality of the auROC (“**roccomp**” command of STATA V.10). Internal consistency analyses were performed using the C-α and hierarchical McDonald's Omega coefficient using maximum likelihood estimation for polychoric variables [58]. Confidence intervals (CI) were calculated at 95% and a p value <0.05 was considered significant. The software STATA, version 10, was used for all statistical analyses, except for the estimation of the McDonald's Omega coefficient MD, for which the R statistical software version 2.15.1 was used.

### Ethics

Prior to their participation in the study, all persons gave written informed consent. The research protocol was reviewed and approved by the Universidad Peruana Cayetano Heredia Ethics Committee.

## Results

A total of 194 participants were recruited, 70 for the MDE, 63 for OPD and 61 for the NEP group. Only three potential participants on the NEP group refused to participate in the study. Among OPD participants, 22 received a DSM-IV diagnosis of any anxiety disorder, 15 of any psychotic disorder, 17 any mood disorder different than MDE, 3 were diagnosed with any substance use disorder and 6 other DSM-IV diagnoses.

Comparison of age, gender, educational level as well as CGI, CES-D and ZSDS median scores between groups are shown in Table 1. We found statistically significant differences regarding the proportion of women in the depression group and in the severity of symptoms according to the CGI-S and the CES-D and ZSDS scores. The proportion of women was significantly higher in the MDE group than in the OPD and NEP groups (p<0.01) while the scores on the CES-D and ZSDS were also significantly higher (p<0.01) in the MDE than in the OPD and NEP groups. The median of both CES-D and ZSDS on the MDE group (35.5 & 53.5) was statistically different (p<0.01) than on the OPD (20 & 40) and NEP (9 & 33). CGI-S scores were found to be significantly different (p<0.01) between MDE (p50: 4, p25: 4, p75: 5) and OPD (p50: 4, p25: 4, p75: 5) groups.

**Table 1:** General Characteristics of the Sample.

	MDE	OPD	NEP	MDE+OPD	MDE+NEP
	(n = 70)	(n = 63)	(n = 61)	p-value	p-value
Age	32.5	32	34	0.64*	0.95*
median (p25; p75)	(24;47)	(23;41)	(24;46)		
Female (%)	71.43	50.79	42.62	0.01†	<0.01†
**Educational Level (%)**				0.46‡	0.05‡
Incomplete Elementary School	10	1.59	1.64	---	---
Complete Elementary School	8.57	14.29	19.67	---	---
Complete High School	41.43	36.51	40.98	---	---
Technical Studies	14.29	15.87	8.2	---	---
University-level Studies	25.71	31.75	29.51	---	---
CGI	4	4	---	<0.01*	---
Median (p25; p75)	(4;5)	(3;4)			
CES-D	35.5	20	9	<0.01*	<0.01*
Median (p25; p75)	(29;43)	(12;27)	(4;16)		
ZSDS	53.5	40	33	<0.01*	<0.01*
Median (p25; p75)	(47;58)	(34;48)	(30;39)		

MDE: Group with Major Depressive Episode; OPD: Group with Other Psychiatric Disorders; NEP: Group with no Psychiatric Disorders; CGI: Clinical Global Impression Scale; CES-D (Center for Epidemiological Studies Depression Scale); ZSDS (Zung Self-Rated Depression Scale).

* Kruskall Wallis Test.

‡ : Fisher exact test.

For the CES-D and ZSDS scales, C-α was 0.93 and 0.89 respectively. Inter-item covariance, item-test and item-rest correlation as well as the alpha change in the absence of each item for both scales, as shown in Table 2. For CES-D Item-test correlation varied from 0.36 (item 4) to 0.3 (item 18), item-rest correlation ranged from 0.29 to 0.81, while the average inter-item covariance from 0.50 to 0.45, with the items 18 and 4 being the best and worst in performance; C-α would drop from 0.93 to 0.92 if items 6 and 18 would be removed. In the ZSDS, item-test correlation varied from 0.18 to 0.77, item-rest correlation from 0.10 to 0.73, average inter-item covariance from 0.26 to 0.30, with item 18 being having the most favorable estimates and item 2 the poorest performance; C-α would improve from 0.89 to 0.90 if item 2 would be retired. Hierarchical McDonald's Omega coefficients were estimated to be 0.90 and 0.75 for the CES-D and ZSDS respectively.

**Table 2:** Item-test, Item-rest correlation, average inter-item covariance and alpha in absence of the Item for the CES-D and ZSDS.

CES-D					ZSDS				
Item	Item-Test Correlation	Item-Rest Correlation	Average Inter-Item Covariance	Cr-α in absence of item	Item	Item-Test Correlation	Item-Rest Correlation	Average Inter-Item Covariance	Cr-α in absence of item
1. I was bothered by things that usually don't bother me.	0.57	0.52	0.48	0.93	1. I feel down-hearted and blue	0.76	0.72	0.26	0.88
2. I did not feel like eating; my appetite was poor.	0.51	0.46	0.49	0.93	2. Morning is when I feel the best	0.18	0.10	0.30	0.90
3. I felt that I could not shake off the blues even with help from my family or friends.	0.80	0.77	0.46	0.93	3. I have crying spells or feel like it	0.63	0.58	0.27	0.88
4. I felt I was just as good as other people.	0.36	0.29	0.50	0.93	4. I have trouble sleeping at night	0.61	0.55	0.27	0.89
5. I had trouble keeping my mind on what I was doing.	0.58	0.52	0.48	0.93	5. I eat as much as I used to	0.40	0.31	0.28	0.89
6. I felt depressed.	0.82	0.79	0.46	0.92	6. I still enjoy sex	0.45	0.36	0.28	0.89
7. I felt that everything I did was an effort.	0.52	0.46	0.48	0.93	7. I notice that I am losing weight	0.40	0.33	0.28	0.89
8. I felt hopeful about the future.	0.61	0.56	0.48	0.93	8. I have trouble with constipation	0.48	0.41	0.28	0.89
9. I thought my life had been a failure.	0.72	0.68	0.46	0.93	9. My heart beats faster than usual	0.52	0.46	0.28	0.89
10. I felt fearful.	0.74	0.71	0.46	0.93	10. I get tired for no reason	0.60	0.54	0.27	0.89
11. My sleep was restless.	0.65	0.60	0.47	0.93	11. My mind is as clear as it used to be	0.68	0.62	0.26	0.88
12. I was happy.	0.71	0.67	0.47	0.93	12. I find it easy to do the things I used to	0.66	0.60	0.27	0.88
13. I talked less than usual.	0.55	0.50	0.49	0.93	13. I am restless and can't keep still	0.52	0.45	0.27	0.89
14. I felt lonely.	0.79	0.76	0.45	0.93	14. I feel hopeful about the future	0.66	0.60	0.27	0.88
15. People were unfriendly.	0.59	0.54	0.48	0.93	15. I am more irritable than usual	0.63	0.57	0.27	0.88
16. I enjoyed life.	0.72	0.68	0.46	0.93	16. I find it easy to make decisions	0.60	0.54	0.27	0.89
17. I had crying spells.	0.70	0.66	0.47	0.93	17. I feel that I am useful and needed	0.71	0.67	0.26	0.88
18. I felt sad.	0.83	0.81	0.45	0.92	18. My life is pretty full	0.77	0.73	0.26	0.88
19. I felt that people dislike me.	0.58	0.54	0.48	0.93	19. I feel that others would be better off if I were dead	0.48	0.42	0.28	0.89
20. I could not get “going.”	0.77	0.74	0.46	0.93	20. I still enjoy the things I used to do	0.73	0.68	0.26	0.88

CES-D: Center of Epidemiological Studies Depression Scale, ZSDS: Zung Self Rating Depression Scale, Cr-α: Cronbach's Alpha.

**Figure 1:**
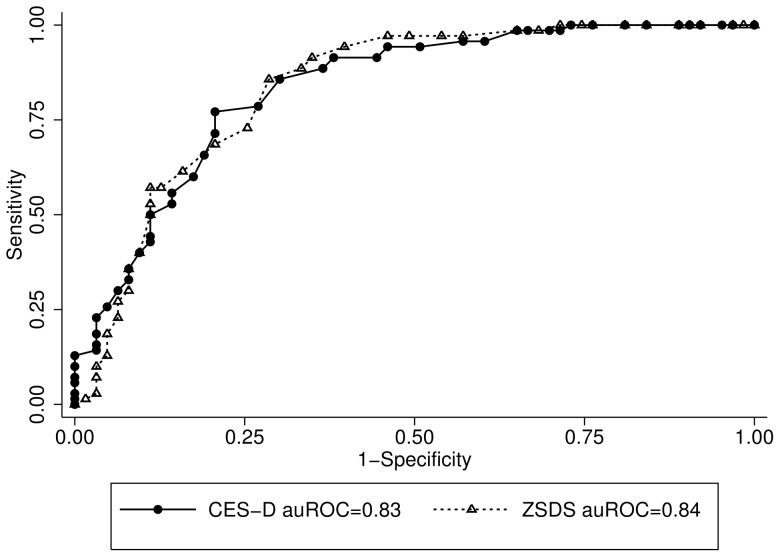
ROC Curves for CES-D (•) and ZSDS (Δ) in the MDE+OPD group.

**Figure 2:**
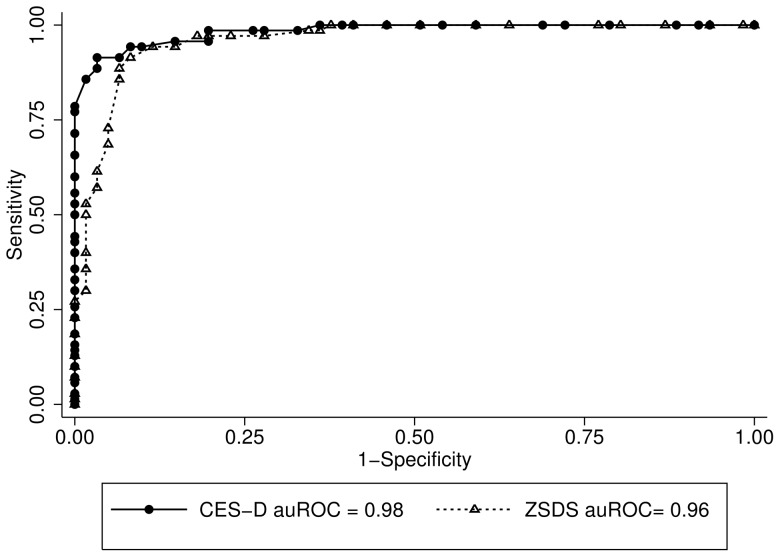
ROC Curves for CES-D (•) and ZSDS (Δ) in the MDE+NEP group.

In the MDE+OPD, the auROC was 0.83 (95% Confidence Interval (CI): 0.76–0.90) for the CES-D and 0.84 (95% CI: 0.76–0.91) for the ZSDS (Figure 1). When we compared the MDE+NEP, the area under the curve was 0.98 (95% CI: 0.97–0.99) for the CES-D and 0.96 (95% CI: 0.93–0.99) for the ZSDS (Figure 2). The CES-D and ZSDS showed good sensitivity and specificity; however the best cut-off scores for the MDE+OPD were higher for both the CES-D (≥29) and ZSDS (≥47), when compared with the cut-off points for the MDE+NEP (≥24 and ≥45, respectively; see Tables 3 and 4).

**Table 3:** Sensitivity, specificity and percentage of correctly classified subjects for CES-D Cut-off scores.

	MDE+OPD			MDE+NEP		
Cut-off Score	Sensitivity	Specificity	Correctly Classified	Sensitivity	Specificity	Correctly Classified
≥18	95.7%	39.7%	69.2%	95.7%	80.3%	88.6%
≥19	95.7%	42.9%	70.7%	95.7%	85.3%	90.8%
≥20	94.3%	49.2%	72.9%	94.3%	90.2%	92.4%
≥21	94.3%	54.0%	75.2%	94.3%	91.8%	93.1%
≥23	91.4%	55.6%	74.4%	91.4%	93.4%	92.4%
≥24	91.4%	61.9%	77.4%	**91.4%**	**96.7%**	**93.9%**
≥25	88.6%	63.5%	76.7%	88.6%	96.7%	92.4%
≥26	85.7%	69.8%	78.2%	85.7%	98.4%	91.6%
≥27	78.6%	73.0%	75.9%	78.6%	100.0%	88.6%
≥29	**77.1%**	**79.4%**	**78.2%**	77.1%	100.0%	87.8%
≥30	71.4%	79.4%	75.2%	71.4%	100.0%	84.7%

MDE: Group with Major Depressive Episode; OPD: Group with Other Psychiatric Disorders; NEP: Group with no Psychiatric Disorders.

**Table 4:** Sensitivity, specificity and percentage of correctly classified subjects for ZSDS Cut-off scores.

	MDE+OPD			MDE+NEP		
Cut Off Score	Sensitivity	Specificity	Correctly Classified	Sensitivity	Specificity	Correctly Classified
≥40	97.10%	46.00%	72.90%	97.10%	77.10%	87.80%
≥41	97.10%	50.80%	75.20%	97.10%	80.30%	89.30%
≥42	97.10%	54.00%	76.70%	97.10%	82.00%	90.10%
≥43	94.30%	60.30%	78.20%	94.30%	85.30%	90.10%
≥44	94.30%	60.30%	78.20%	94.30%	88.50%	91.60%
≥45	91.40%	65.10%	79.00%	**91.40%**	**91.80%**	**91.60%**
≥46	88.60%	66.70%	78.20%	88.60%	93.40%	90.80%
≥47	**85.70%**	**71.40%**	**78.90%**	85.70%	93.40%	89.30%
≥48	72.90%	74.60%	73.70%	72.90%	95.10%	83.20%
≥49	68.60%	79.40%	73.70%	68.60%	95.10%	80.90%
≥50	61.40%	84.10%	72.20%	61.40%	96.70%	77.90%

MDE: Group with Major Depressive Episode; OPD: Group with Other Psychiatric Disorders; NEP: Group with no Psychiatric Disorders.

No statistically significant differences were found between the area under the ROC curve in the CES-D and ZSDS for the MDE+OPD (p = 0.94) or the MDE+NEP (p = 0.14) groups.

We also found a good correlation between the ZSDS and CES-D scores (rho = 0.86, p<0.001), and a statistically significant correlation was also found between the CES-D and CGI scores (rho = 0.51, p<0.001), as well as for the ZSDS and CGI scores (rho = 0.50, p<0.001).

## Discussion

We studied the psychometric properties of the ZSDS and CES-D in two contexts, one in which we analyzed the performance of the scales in a general hospital population (MDE+NEP) and one in which major depression has to be detected in a setting with high prevalence of other psychiatric disorders (MDE+OPD).

### Internal Consistency and Discriminant Validity

Cronbach's Alpha results for both scales (CES-D = 0.93, ZSDS = 0.89) were consistent with reports for the CES-D both in a Spanish-language study in a population with affective disorders (0.90) [29] and also with the internal consistency of the DPS (0.86) which was validated on the PNMHI, with a similar population [16]. Studies with non-general hospital or specialized health care center report internal consistency estimations ranging from 0.85 to 0.93 for the CES-D in Spain [25], [27], 0.80 for a 10 item Colombian version of the ZSDS and 0.85 for the full scale in Puerto Rico general population [31], [33]. The McDonald's Omega coefficient (CES-D = 0.90, ZSDS = 0.75) has been proposed as a more appropriate alternative to Cronbach's Alpha as index of how items on an instrument measure the same latent variable [59], [60]; however, the availability of literature to compare the performance of the studied scales in different settings is limited.

On the MDE+OPD group comparisons, sensitivity and specificity (∼80%) was below the results reported by *Soler et al* (ss and sp ∼90%) and a little above to the findings on the DPS validation (ss ∼77%; sp ∼72%) when using the clinical diagnosis of a psychiatrists as gold standard [16], [29]. The difference found with the study of *Soler et al*, might be the result of the use of a different gold standard, in this case the HRSD. On the MDE+NEP group, sensitivity and specificity for both scales was ∼92%, which appears to be slightly above the findings on other Latin America studies in general population for both the CES-D and the ZSDS (ss = 73–95.5%, sp = 70.7–70.4%) [25]–[27], [31], [33].

In previous studies including non-Spanish validations, the recommended cut-off score for the CES-D has varied among populations, from ≥12 to ≥21 for clinically significant depressive symptoms [61]–[63] and from ≥23 to 26 for major depression [38], [40], [64], [65]. As the clinical assessment by psychiatrists according to DSM-IV criteria was considered the gold standard for MDE diagnosis, our results (co≥24) are fairly consistent. For the ZSDS, the best cut-off score (co≥45) was similar to the results for the Greek validation of the ZSDS [52]. The recommended cut-off scores for the MDE+OPD groups were higher (CES-D: ≥29; ZSDS; ≥47) and with lower sensitivity and specificity than the ones for the MDE+NEP groups (Tables 3 and 4).

No statistical differences (p<0.05) were found in the area under the ROC curve comparison between the CES-D and the ZSDS in the MDE+NEP or the MDE+OPD groups. These findings suggest that both scales could have the same predictability of depression between the studied groups, and are valid and consistent psychometric tools in both a general hospital and high prevalence of mental disorders settings, although discriminative properties seems to be diminished for the latter group, as discussed below.

### Variation of discriminative properties in non-population settings

Evidence suggests that comorbidity with pathologies with symptoms similar or related to depressive syndromes might affect the performance of psychometric instruments. In studies with patients suffering of rheumatoid arthritis, seven items (items number 2, 4, 8, 11, 12, 16 and 18) had to be removed from the CES-D to reach a good fit for the scale; once retired, the 13 item scale showed a ss = 89.6% and sp = 95.8%, with a co≥9 [66]. The influence of somatic components affecting rheumatoid arthritis, regardless of presenting or not MDE was also supported by *Callahan et al* [67]. In another study that compared depressive symptoms using the ZSDS in a group of patients with chronic pain and patients from psychology service with comparable depressive symptoms, chronic patients endorsed items referring to psychomotor retardation, sleep disturbance, constipation and fatigue, and thus, might overestimate the depressive symptoms in a similar sample [68]. In a sample of patients suffering from multiple sclerosis, the cut-off score of 16 on the CES-D, which is recommended as cut-off score for detection of clinically significant depressive symptoms, yielded low positive predictive values both for any depressive disorder (74.5%) and major depressive disorder (59.6%), thus suggesting that the score used for general population might not be appropriate for this particular setting [69]. These studies suggest that somatic symptoms that present similitudes to those characteristic of depressive disorders might have a negative effect on the performance of both scales, and also increase their optimal the cut-off scores. Taking this into account, symptoms common with major depression found in various psychiatric disorders could diminish the discriminate properties of the scales, as evidenced by the similar findings of discriminative properties of CES-D and ZSDS described in this study and the DPS validation, which found similar values in similar, even if not identical settings [16].

### Limitations

The main limitation for this study was that the gold standard for the diagnosis of major depression was not a standardized interview but clinical diagnosis by a psychiatrist. Further, the inter observer concordance for the clinical DSM-IV based diagnosis could not be assessed. This and the possible re-arrangement of items for a high prevalence of mental disorder settings should be engaged in future studies. The results of this study cannot be generalized to other samples that do not meet the conditions and characteristics of this specific setting.

With respect to the use of gold standard, *Vega-Dienstmaier et al* used both a clinical interview (SCID) and clinical diagnosis of a psychiatrist as gold standard for the validation of the DPS. The auROC was slightly higher when using the SCID as gold standard (0.87 vs. 0.83); the same was true for sensitivity (81.3% vs. 77.7%) and specificity (80% vs. 72.3%). The optimal cut-off score was 1 point lower when using the clinical diagnosis as gold standard (≥27 vs. ≥26) [16]. While the psychometric estimates appear to favor the SCID, the study does not report if this difference is statistically significant different, particularly in the auROC for both assessments. In general, studies validating Spanish versions of depression screening instruments used as gold standards structured interviews [26], [27], clinical diagnosis of MDE [18], [30], or both [16]. The most reasonably approach might be to collect information of both clinical and structured interview based diagnosis during future evaluations of psychometric tools.

While the psychometric properties of both CES-D and ZSDS in the MDE+OPD group appears to be different than reported performance on general population settings; on the MDE and NEP group, both cut-off score and discriminant properties appears to be congruent with estimates calculated based on general population data.

### Use of Results and Further Studies

The availability of validated screening instruments such as the CES-D and ZSDS might represent an important contribution to both research and screening of MDE. This becomes especially important if we take into account that an important amount of sub-diagnosis of depressive disorders in primary care settings of developing countries has been reported [35] and that very limited resources for independent research have been identified as obstacles to proper mental health policies supported by solid scientific evidence [70], [71]. The amount of reported interventions focusing on prevention, treatment and rehabilitation of important contributors to burden of disease, such as depressive disorders, are still scarce in Latin America [72]. We expect that the availability of free to use instruments will encourage independent research focused on depressive disorders, as interventions designed in more developed countries might not be useful in Latin American or other developing settings [73].

Factor structure, test-retest reliability and general population estimates of the psychometric properties of both CES-D and ZSDS might be considered as target for research. Alternative forms of these instruments, such as shorter, telephone or online versions might also be useful in facilitating independent research in developing countries.

We conclude that both CES-D and ZSDS are reliable and consistent instruments for detection of MDE in psychiatric and general hospital settings.
